# Spatial distribution, *Leishmania* species and clinical traits of Cutaneous Leishmaniasis cases in the Colombian army

**DOI:** 10.1371/journal.pntd.0005876

**Published:** 2017-08-29

**Authors:** Luz H. Patino, Claudia Mendez, Omaira Rodriguez, Yanira Romero, Daniel Velandia, Maria Alvarado, Julie Pérez, Maria Clara Duque, Juan David Ramírez

**Affiliations:** 1 Grupo de Investigaciones Microbiológicas-UR (GIMUR), Programa de Biología, Facultad de Ciencias Naturales y Matemáticas, Universidad del Rosario, Carrera 24# 63C-69, Bogotá, Colombia; 2 Laboratorio de Referencia e Investigación en Enfermedades Tropicales, Dirección de Sanidad Ejército, Ejército Nacional de Colombia. Avenida Carrera 7 No 52–48. Bogotá, Colombia; Saudi Ministry of Health, SAUDI ARABIA

## Abstract

In Colombia, the cutaneous leishmaniasis (CL) is the most common manifestation across the army personnel. Hence, it is mandatory to determine the species associated with the disease as well as the association with the clinical traits. A total of 273 samples of male patients with CL were included in the study and clinical data of the patients was studied. PCR and sequencing analyses (Cytb and HSP70 genes) were performed to identify the species and the intra-specific genetic variability. A georeferenced database was constructed to identify the spatial distribution of *Leishmania* species isolated. The identification of five species of *Leishmania* that circulate in the areas where army personnel are deployed is described. Predominant infecting *Leishmania* species corresponds to *L*. *braziliensis* (61.1%), followed by *Leishmania panamensis* (33.5%), with a high distribution of both species at geographical and municipal level. The species *L*. *guyanensis*, *L*. *mexicana* and *L*. *lainsoni* were also detected at lower frequency. We also showed the identification of different genotypes within *L*. *braziliensis* and *L*. *panamensis*. In conclusion, we identified the *Leishmania* species circulating in the areas where Colombian army personnel are deployed, as well as the high intra-specific genetic variability of *L*. *braziliensis* and *L*. *panamensis* and how these genotypes are distributed at the geographic level.

## Author summary

Colombia is one of the countries with the highest incidence of Cutaneous Leishmaniasis in the world and the army population is the most vulnerable population. Herein, we identified the infecting *Leishmania* species *(L*. *braziliensis*, *L*. *panamensis*, *L*. *guyanensis*, *L*. *mexicana* and *L*. *lainsoni*). We also showed the high intra-specific genetic variability of *L*. *braziliensis* and *L*. *panamensis* and how these genotypes are distributed at the geographic level.

## Introduction

Leishmaniasis is a group of tropical diseases caused by parasites of the genus *Leishmania*. This parasite is transmitted to humans through the bite of infected female insects of the family Psychodidae.[1, 2] There are about 20 species of *Leishmania*, which can cause different clinical manifestations in humans, including Cutaneous Leishmaniasis (CL), Mucocutaneous Leishmaniasis (ML) and Visceral Leishmaniasis (LV).[2, 3] These diseases are a major public health problem in 98 countries around the world, where 12 million people are infected, more than 350 million people at risk of infection and 1.3 million new cases per year occur.[2] CL is the most common clinical manifestation; between 0.7 and 1.3 million new cases of CL are reported annually and about 90% of them occur in countries such as Afghanistan, Algeria, Brazil, Iran, Syria and Colombia.[2, 3]

Colombia occupies the second place in incidence of Leishmaniasis in America after Brazil.[4] In 2016, about 10,743 new cases of Leishmaniasis were reported in the national territory (2,493 more than the year immediately preceding), of which 98% were associated with CL.[5] The increase in the number of cases has been attributed to different factors such as the colonization of vectors into new geographical areas, the urbanization of the disease [4] and the human activities that expose immune populations to infection, such as traveling, migration, civil conflict and military operations. [4, 6–8] Regarding the latter, the army population shows the highest incidence of the disease and constitute the most vulnerable group, due to the continuous deployment of troops to areas of high endemicity and high circulation of the insect vector.[8] Several CL outbreaks in the army population have been reported in different countries of the world including Colombia.[4, 9–12] These outbreaks not only affect the foot of force, the cessation of military operations, the quarantine throughout the battalion in which the disease occurs, but also the risks to which the military must be subjected by the use of anti-leishmanial treatments (which have a high degree of toxicity).

Epidemiological data generated by the Public Health Surveillance System of the Military Health Service of Colombia (Salud Operacional DISAN Ejercito) report a high number of cases associated with Leishmaniasis during the years 2011 to 2017 (17.796 cases of CL and 254 cases of MCL). However, very few studies report circulating species. The most recent study, conducted in a small army population (43 individuals), describes that *Leishmania (Viannia) braziliensis* is the predominant species (95.4%), followed by *Leishmania (Viannia) guyanensis* (2.3%).[13] Contrary to what was reported in populations living in urban areas where the most frequent species are *Leishmania (Viannia) panamensis* (61.3%) and *Leishmania (Viannia) braziliensis* (27.1%).[14] In spite of the existing data, to date there are no studies where using a larger army population are described the main species of *Leishmania* as well as the geographical distribution within the national territory. Similarly, there are very few studies reporting the genetic variability of the infecting species in relation to the geographical distribution.

Therefore, the objectives of this study were to evaluate the clinical traits, distribution and genetic variability of *Leishmania* species that circulate in the areas where army personnel are deployed using clinical samples (imprint in filter paper) from patients with CL.

## Materials and methods

### Study population

A total of 273 samples from male patients belonging to the Colombian National Army were collected. The selection of the patients was made by selective and stratified sampling and carried out on individuals with positive diagnosis of CL in 12 army units located throughout the country (which reported the major number of cutaneous leishmaniasis cases during 2013).The collection of the samples was pretty accurate at department level (Colombian administrative subdivisions). Sampling was stratified by the number of cases reported by each unit divided by the total number of cases.

As inclusion criteria, patients had to be male, over 18 years of age, with clinical and parasitological diagnosis of CL; with lesions of minimum one month and maximum three months of evolution and without anti-leishmanial treatment for at least two months prior sampling. Only patients with a positive result for at least one direct smear or PCR-skin biopsy were included in the study. Those patients with lesions in face, genitals or mucosa and secondary infected lesions were not sampled. A summary of the demographic and clinical characteristics of patients included in this study are presented in the **Table 1**.

**Table 1:** Demographic and clinical characteristics of patients.

Characteristics	N: 221	Estimate	95%, CI
**Age, median (p25-p75)**	23	(22–26)	23–24
**Skin type**			
Brown	109	49.3%	42.5–56.1
White	96	43.4%	36.7–50.2
Black	11	4.9%	1.88–8.07
ND	5	-	-
**Infected lesion**	34	15.4%	10.4–20.4
**Previous leishmaniasis**	41	19.5%	14.1–24.9
Cutaneous	40/41	97.5%	80.9–98.5
Mucocutaneous	1/41-	2.5%	0.06–12.3
**Scar**	33/41	80.5%	69.5–95.5
Head/Neck	8/33-	24.2%	8.11–40.4
Trunk	5/33-	15.2%	5.11–31.9
Upper limbs	20/33	60.6%	42.4–78.8
Lower limbs	9/33-	27.3%	10.6–43.9
**Actual leishmaniasis**			
Cutaneous	220	99.6%	97.5–99.9
Mucocutaneous	1	0.45%	0.01–2.49
**Species associated with the actual leishmaniasi**s			
*L*. *braziliensis*	135	61.1%	54.4–67.7
*L*. *panamensis*	74	33.5%	27.0–39.9
*L*. *guyanensis*	8	3.6%	0.93–6.30
*L*. *mexicana*	3	1.4%	0.28–3.91
*L*. *lainsoni*	1	0.4%	0.01–2.49

**Age:** expressed in years, **ND:** not data.

### Ethics statement and sample collection

Once the adult patients accepted their participation in the study and after reading and signing written informed consent, a survey was conducted which included information associated with demographic factors (age, place of birth, site of possible acquisition of Leishmaniasis, personal protection measure to avoid insect bite (use of mosquito repellents, repellents and/or uniform use impregnated with Permethrin) and clinical factors (previous Leishmaniasis, clinical presentation, number and diameter of lesions of current leishmaniasis, time of evolution of the disease, lymphadenopathy and previous anti-leishmanial treatment)). Subsequently, the samples were obtained by imprint in filter paper of the site of the lesion (in order to completely cover a quarter of filter paper per patient); the numbers of imprints taken varied from patient to patient according to the size of the lesion. The imprints were stored at 4°C until use. Once the samples were taken, all patients were treated with meglumine antimoniate (Glucantime), according to Colombia’s treatment guide for Leishmaniasis.

All protocols applied in the study were approved by the Ethics Committee of the Central Military Hospital of Colombia, in accordance with the principles established in the Declaration of Helsinki and Resolution 008430 of October 4, 1993 of the Ministry of Health.

### DNA extraction and PCR amplification for the identification of *Leishmania* species

DNA extraction was performed using the commercial kit ISOLATE II Genomic DNA kit (Bioline), following the protocol described by the manufacturer. Subsequently, species identification was performed using direct sanger sequencing of the genes coding for the cytochrome b (Cytb) molecules and the heat shock protein (HSP70), as described by Ramírez et al, 2016 [14] and Hernandez et al., 2014. [15] The PCR reaction was carried out at a final volume of 25 μl containing 12.5 μl GoTaq Green Master Mix (Promega, Madison, Wi, US), 0.5 μM of each primer and 1.25 μL DNA (Concentration <250 ng). PCR conditions for the amplification of the Cytb gene are described below: Initial denaturation at 95°C for 5 min, 40 denaturation cycles at 95°C for 1 min, annealing at 58°C for 1 min, extension at 72°C for 1 min, and final extension at 72°C for 10 min. For the HSP70 gene, the following conditions were used: initial denaturation 94°C for 5 min, 40 denaturation cycles at 94°C for 1 min, annealing at 60°C for 1 min, extension at 72°C for 1 min and Final extension at 72°C for 10 min. Amplification and size of the amplicon was verified by agarose gel electrophoresis stained with SYBR Safe DNA Gel Stain (Life Technologies, Carlsband, Ca, US) and a molecular weight marker (Promega). The amplification products were purified with EXOSAP (Affymetrix, USA) and sequenced by the dideoxy-terminal method, in an automated capillary sequencer (AB3730, Applied Biosystem). Subsequently the sequences were subjected to BLASTn to search similarity with the *Leishmania* sequences deposited on GenBank. [14]

### Phylogenetic reconstruction, haplotype and nucleotide diversity analyses

The sequences obtained were edited in MEGA 5.0 and aligned with CLUSTAL W, using the reference sequences for the Cytb gene of *Leishmania donovani donovani* (AB095957), *Leishmania garnhami* (AB095965), *Leishmania mexicana* (AB095936), *Leishmania amazonensis* (AB095964), *Leishmania garnhami* (AB095965), *Leishmania mexicana* (AB095960), *Leishmania braziliensis* (AB095966), *Leishmania panamensis* (AB095968), *Leishmania guyanensis* (AB095969), *Leishmania equatoriensis* (AB434687), *Leishamnia pifanoi* (EF579907), *Leishmania lainsoni* (AB433280), *Leishmania colombiensis* (KF302738) and *Leishmania peruviana* (AB433282). For The HSP70 gene the reference sequences of *L*. *major* (HF586403.1), *L*. *donovani* (JX312712.1), *L*. *tropica* (HF586409.1), *L*. *peruvian*a (HF586368.1), *L*. *aethiopica* (HF586411. 1), *L*. *garnhami* (EU599092.1), *L*. *chagasi* (FN395037.1), *L*. *mexicana* (XM 003877072.1), *L*. *amazonensis* (L14605.1), *L*. *braziliensis* (AF291716.1), *L*. *guyanensis* (EU599093.1), *L*. *infantum (chagasi)* (XM 003392632.1), *L*. *panamensis* (FN395055.1) and *L*. *lainsoni* (FN395049.2) were employed. A maximum composite likelihood (MCL) analysis using a Tamura-3 parameter was run in RaxML Phylogeny.fr platform. To evaluate the robustness of the nodes in the resulting phylogenetic tree, 1000 bootstrap replicates were performed. In addition to MCL analyzes, a Nexus matrix was constructed for haplotype network analysis in Network 2.0 using a median-joining model based on 1000 iterations with default parameters. The purpose of this analysis was to determine the number of alleles across the population and determine the biological and geographical distribution of the alleles depicted for two species (*L*. *panamensis* and *L*. *braziliensis*). In order to analyze the distribution of species and haplotypes at the geographic level, the 32 Colombian departments were divided into five eco-geographical regions (Orinoquia, Amazonian, Andean, Atlantic and Pacific). Lastly, sequence genetic diversity was estimated for Cytb and HSP70 genes fragments by the most frequent species set. Π and θ nucleotide diversity indexes and haplotype diversity were calculated in DNAsp v.5.0.

### Spatial distribution patterns

To address the spatial distribution of *Leishmania* parasites isolated, a georeferenced database was constructed. Data on human isolates belong to the posible site of infection acquisition, as reported by the patient. Using ArcGIS10.3 we extracted values of Ecoregions [16] and Colombian Ecosystems [17] in order to describe parasite distribution by Ecosystems and land´s use coverage.

### Statistical analyses

The relationship between the clinical-demographic variables and the infecting *Leishmania* species was analyzed using the statistical packages XLSTAT (Version 2014.5.03), Minitab (Version 17) and SPSS Modeler (Version 18). Quantitative data were expressed in medians, qualitative in proportions (95% CI), the confidence interval to proportion (CI95%) was calculated using the next equations: 1. Upper limit of confidence Interval, P+ 1.96*(√p(1-p)/n). 2. Lower limit of confidence Interval, P- 1.96*(√p(1-p)/n]) and comparisons between variables were performed with non-parametric statistics. A p value <0.05 was established to determine statistical significance. With a mesh chart (SPSS Version 18) we explored the strength of relationships between species of *Leishmania* and specific characteristics that previously demonstrated a statistically significant relationship (Chi squared, X2).

## Results

### Identification of *Leishmania* species and phylogenetic reconstruction

**Fig 1:**
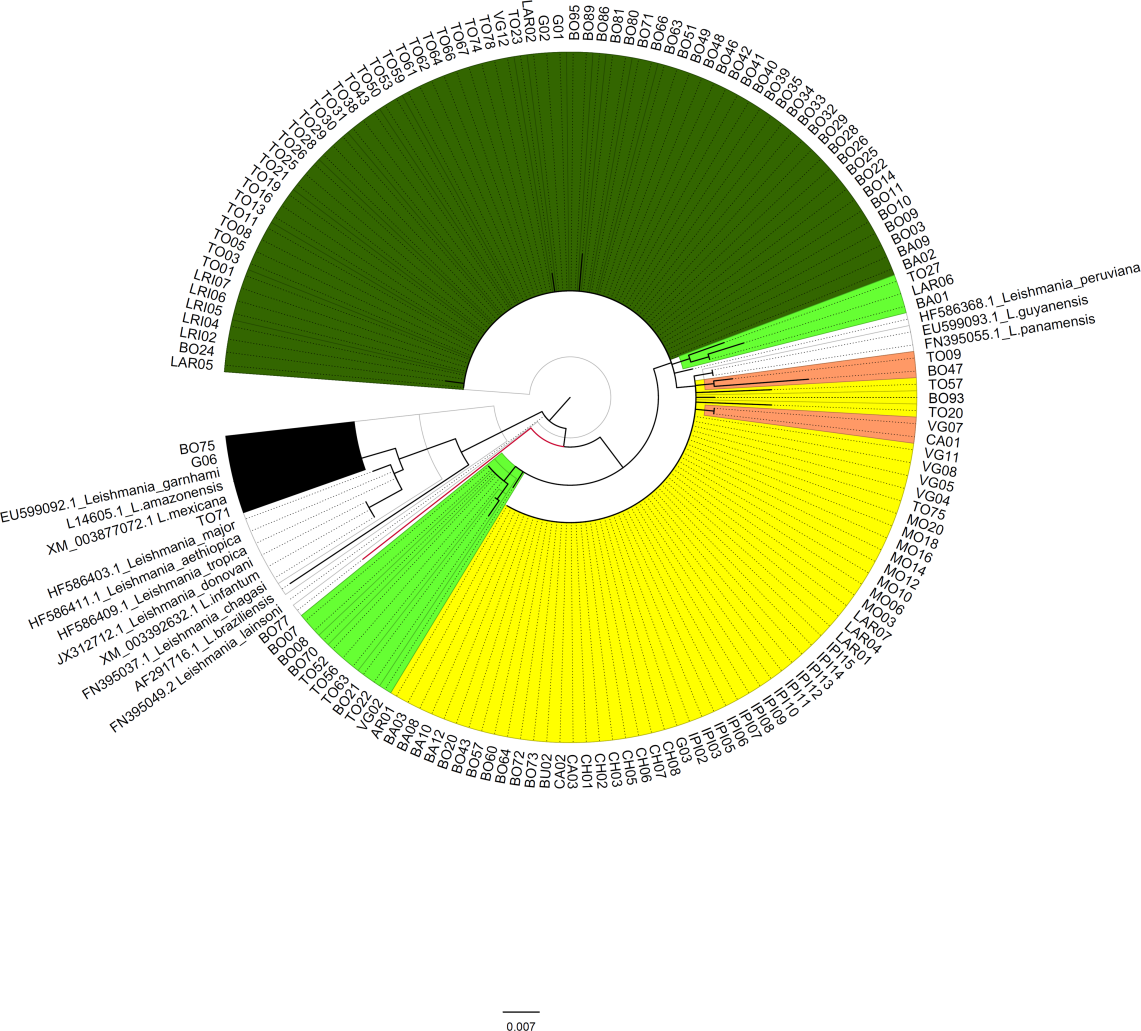
Phylogenetic reconstruction of HSP70 sequences. Phylogenetic reconstruction of 150 HSP70 gene sequences obtained from direct samples of CL patients and the reference strain retrieved from GenBank. *L*. *braziliensis* (green); *L*. *panamensis* (yellow); *L*. *mexicana* (black) and *L*. *lainsoni* (red). Futhermore, different genotypes associated with *L*. *braziliensis* (light green) and *L*. *panamensis* (light pink) were identified. The numbers represent the coding of each sample.

Sequencing analysis performed on a fraction of the Cytb and HSP70 genes allowed the identification of *Leishmania* species in 221 of the 273 samples analyzed (81%). Seventy-one of them were identified with Cytb and 150 with HSP70. The remaining 52 samples could not be analyzed due to the low amount of DNA of the parasite present in the sample. The 221 sequences edited, were submitted to Blastn, in search of similarity with the sequences deposited on the Genebank database. In general, the sequences had an average identity of 97%. *Leishmania braziliensis* (61.1%, 95% CI, 54.4–67.7), *Leishmania panamensis* (33.5%, 95% CI, 27.0–39.9), *Leishmania guyanensis* (3.6%, IC95%, 0.93–6.30), *Leishmania mexicana* (1.4%, IC95%, 0.28–3.91), and *Leishmania lainsoni* (0.4%, IC95%, 0.01–2.49), were identified in the final consensus of the Cytb and HSP70 typing (**Table 1**). The sequences obtained from the HSP-70 gene were aligned and a robust phylogenetic reconstruction was carried out (Boostrap greater than or equal to 85%). The results allowed the identification of four fully differentiated clusters corresponding to *L*. *braziliensis*, *L*. *panamensis*, *L*. *mexicana* and *L*. *lainsoni*, confirming the results obtained. It was also possible to show that within the clusters of *L*. *braziliensis* and *L*. *panamensis*, different genotypes occur (**Fig 1**).

### Clinical and epidemiological traits

We analyzed 221/273 records of patients with median age (p25-p75) 23 years (22–26), mostly brown skin (49.3%). Nineteen point five percent (95% CI, 14.1–24.9) of the patients reported having previously presented leishmaniasis, of which 97.5% were associated with CL, the 80.5% (95% CI, 69.5–95.5%) of them presented sequelae of scars with a predominantly upper extremity compromise (60.6%, 95% CI, 42.4–78.8) (**Table 1**). The majority of patients (93%) were treated with meglumine antimoniate (mean dose of 20 mg antimony / kg / day), with a duration between 2–45 days of treatment (median of 20 days). The patients who received the treatment for less or more time than the standardized protocol (twenty days) was due to interruption of drug administration as consequence of altered clinical exams that compromise patient safety and health or due to more than one treatment cycle for an unfavorable response. Finally, we identified a 31.67% (95%CI, 19.06–44.27) of new ocurrence of leishmaniasis (probably associated with resistance to treatment, reactivation or reinfection). This value was calculated according to the data generated of the visual confirmation of scars and the described by the patients (which may not be very conclusive).

Regarding the current disease, it was observed that despite the use of personal protection elements to avoid insect bites (repellents, insect repellents or Permethrin impregnated uniform), all the patients included in the study presented clinical or epidemiological criteria positive for Leishmaniasis. Clinical evaluation criteria showed that in more than 95% of the cases, the lesions presented were localized and of cutaneous variety, with the upper extremities being the most affected body region (59.8%, 95% CI, 62.6–66.9%), with a number of lesions ranging from 1–3 (95% of patients), of which 50% had a diameter of 1.2 cm^2^ (p75, 3 cm^2^).

**Fig 2:**
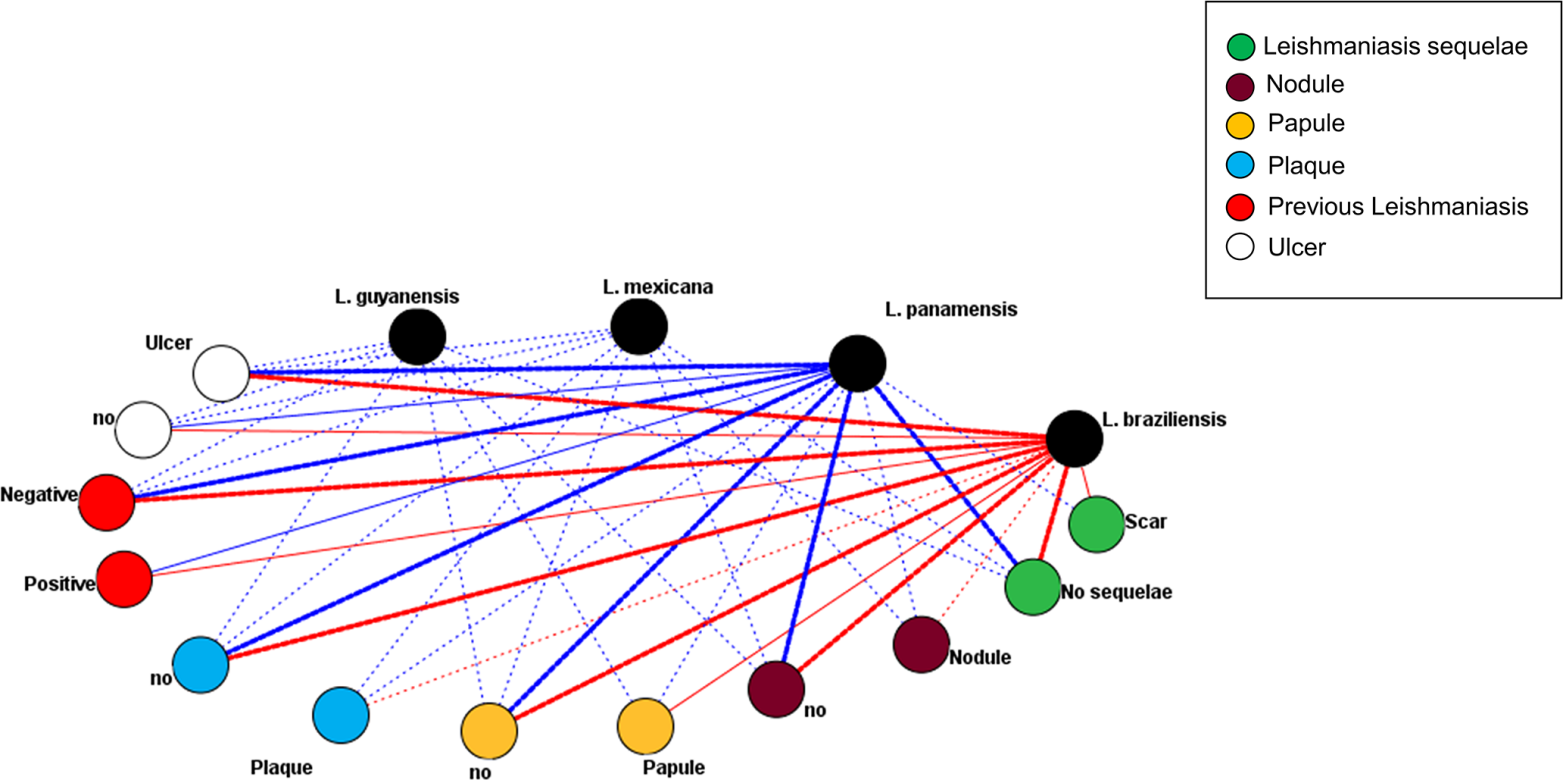
Relationship between clinical-epidemiological data and infecting *Leishmania* species. The frequency of linkages between species of *Leishmania* and the specific characteristics were divided into three levels: **1.** Thickness lines,> 35 links, **2.** Intermediate thickness lines, 15–35 links and **3.** Semi-dotted lines of weak thickness, <15 links. The links with *L*. *braziliensis* were highlighted in red because of the high frequency of identified cases. “no”: it refers to absence of clinical manifestation.

On the other hand, we evaluated the relationship between the collected clinical-epidemiological data and the infecting *Leishmania* species. We identified that certain parameters had a strong relationship with the *Leishmania* species. The results obtained in the mesh graph showed the strong relationship between *L*. *braziliensis* and *L*. *panamensis* with the absence of previous leishmaniasis and recurrent lesions, and with the clinical presentation (strong association with ulcerative and weak lesions with papular, nodular lesions and the presence of plaques (defined as an elevated lesion of the skin of more than 2 centimetres in diameter formed by the union of several papules or nodules)). Contrary to what happened with the other species identified, where a weak association with the evaluated parameters was observed **(Table 2; Fig 2)**.

**Table 2:** Relationship between the clinico-epidemiology features and the infectious *Leishmania* species.

Variables	*L*. *braziliensis* n: 135	*L*. *panamensis* n: 74	*L*. *guyanensis* n: 8	*L*. *mexicana* n: 3	*L*. *lainsoni* n: 1	p-value
**History**	26 (19.3)	16 (21.6)	-	1 (33.3)	-	**0.000**
**Scars**	21 (15.6)	14 (18.9)	-	-	-	**0.000**
**Semiology**						
Papule	21 (17.4)	7 (12.3)	1 (16.7)	-	-	0.742
Ulcer	96 (79.3)	38 (66.7)	5 (83.3)	1 (50)	1 (100)	0.187
Nodule	11 (9.1)	8 (14.0)	-	1 (50)	-	0.354
Plaque	4 (3.3)	4 (7.0)	-	1 (50)	-	**0.002**

**NA:** not applicable, **-:** not events. Kruskall Wallis test was used to compare medians (IQR), Chi squared (***X***^***2***^) was used to compare frequencies, **History**: previous leishmaniasis, **Scars**: scars secondary to leishmaniasis infections.

### Nucleotide diversity analyses

The diversity analysis performed for the Cytb gene, in 61 sequences analyzed, showed a total of 315 polymorphic sites and 431 mutations for *L*. *braziliensis*, *L*. *panamensis* and *L*. *guyanensis*. Based on the haplotype (Hd) and nucleotide diversity indexes (π), for each species, *L*. *guyanensis* showed a marked genetic (Hd = 1) and nucleotide diversity (π = 0.20557), contrary to what was observed with *L*. *braziliensis* and *L*. *panamensis* which, despite having a high genetic diversity (Hd = 0.964 and 0.962, respectively) showed a moderate nucleotide diversity (π = 0.03929 and 0.04752, respectively) (**Table 3**). Regarding the HSP70 gene, a total of 38 polymorphic sites and 40 mutations for *L*. *braziliensis*, *L*. *panamensis* and *L*. *mexicana* were observed in 145 analyzed sequences. Haplotypic and nucleotide diversity indexes revealed that *L*. *mexicana* was the species with the highest values (Hd = 0.667, π = 0.00473), compared to the other two *Leishmania* species analyzed **(Table 3).**

**Table 3:** Genetic diversity parameters of *Leishmania* Cytb and HSP70 genes sequences.

**Cytb gene**						
**Species**	**N**	**S**	**Eta**	**Hd**	**π**	**K**
*L*. *braziliensis*	46	204	257	0,962	0,04752	24,379
*L*. *panamensis*	8	77	82	0,964	0,03929	21,179
*L*. *guyanensis*	7	258	305	1	0,20557	99,905
All species	61	315	431	0,978	0,06979	32,521
**HSP70 gene**						
**Species**	**N**	**S**	**Eta**	**Hd**	**π**	**K**
*L*. *braziliensis*	82	14	14	0,33	0,00281	0,787
*L*. *panamensis*	60	15	15	0,25	0,00217	0,597
*L*. *guyanensis*	3	2	2	0,667	0,00473	1,333
All species	145	38	40	0,653	0,00608	1,666

N = Number of sequences. S = Number of polymorphic sites. Eta = Total number of mutations. Hd = Haplotype diversity. π = Nucleotide diversity. K = Average number of nucleotide differences.

### Patterns of spatial distribution

**Fig 3:**
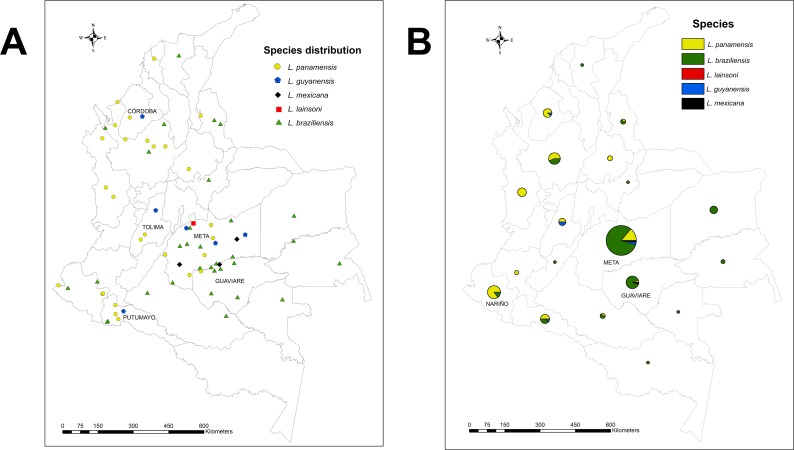
Geographical distribution of 222 *Leishmania* isolates associated to CL in Colombia. GPS coordinates were used to build georeferenced maps of isolates location. The maps were built on ArcGIS10.3 using Esri Colombia PublicadorSIG layer. (http://www.arcgis.com/home/item.html?id=b051fbef7fba406fbb8e62b90925f365#overview) (**A**) Georeferenced isolates discriminated by species in the country. (**B**) Relative abundance of *Leishmania* species in each Colombian department. The size of the circle refers to the number of samples collected by department.

When the analysis of species at the departmental level was made, the patterns of geographic distribution showed that *L*. *braziliensis* and *L*. *panamensis* were distributed differently, while 136 clinical samples of *L*. *braziliensis* were distributed in 14 departments (predominance in the Orinoquia and Amazon regions), 74 samples of *L*. *panamensis* were distributed in 12 departments (with a greater predominance in the pacific region), which reflects the wide geographical distribution of *L*. *panamensis* at national level. Contrary to the other *Leishmania* species identified in the study, whose geographical distribution was limited to certain departments, such as *L*. *guyanensis* distributed in the departments of Meta, Tolima, Putumayo and Córdoba. *L*. *mexicana* in the departments of Meta and Guaviare and *L*. *lainsoni* in the department of Meta **(Fig 3A)**. When we performed the analysis of abundance of species at the departmental level, we observed that the departments with the greatest number of positive samples were Meta, Guaviare and Nariño, of which Meta was the department that provided the largest number of cases of CL in the Colombian army population (48%) during November 2014 to June 2015 (*L*. *braziliensis*: 74/120, *L panamensis*: 14/66, *L*. *guyanensis*: 3/8 and *L*. *mexicana*). In this study, all the species identified (*L*. *braziliensis*, *L*. *panamensis*, *L*. *guyanensis*, *L*. *mexicana and L*. *lainsoni*) were circulating in the Meta department **(Fig 3B)**.

Regarding the distribution of species at the municipal level, we could observe that in some municipalities, two or more species of *Leishmania* are circulating at the same time, as is the case of La Uribe (Meta) in which 4 of the five-species identified circulate (*L*. *braziliensis*, *L*. *panamensis*, *L*. *guyanensis* and *L*. *mexicana*), in Puerto Caicedo (Putumayo) and Vista Hermosa (Meta) are circulating *L*. *braziliensis*, *L*. *panamensis* and *L*. *guyanensis*, in Puerto Lleras (Meta) are circulating *L*. *braziliensis*, *L*. *guyanensis* and *L*. *mexicana*; and in municipalities such as San Jose (Guaviare), Rioblanco (Tolima), Tierra Alta (Córdoba) Apartadó (Antioquia), La Macarena (Meta), Puerto Rico (Brazil), Puerto Cachicamo (San José del Guaviare), Puerto Validibia (Antioquia) and Tumaco (Nariño), are circulating two of the species identified (*L*. *braziliensis* / *L*. *panamensis* or *L*. *braziliensis* / *L*. *mexicana* or *L*. *panamensis* / *L*. *guyanensis*) **(Fig 3B and S1 Fig)**.

### Haplotype networks

**Fig 4:**
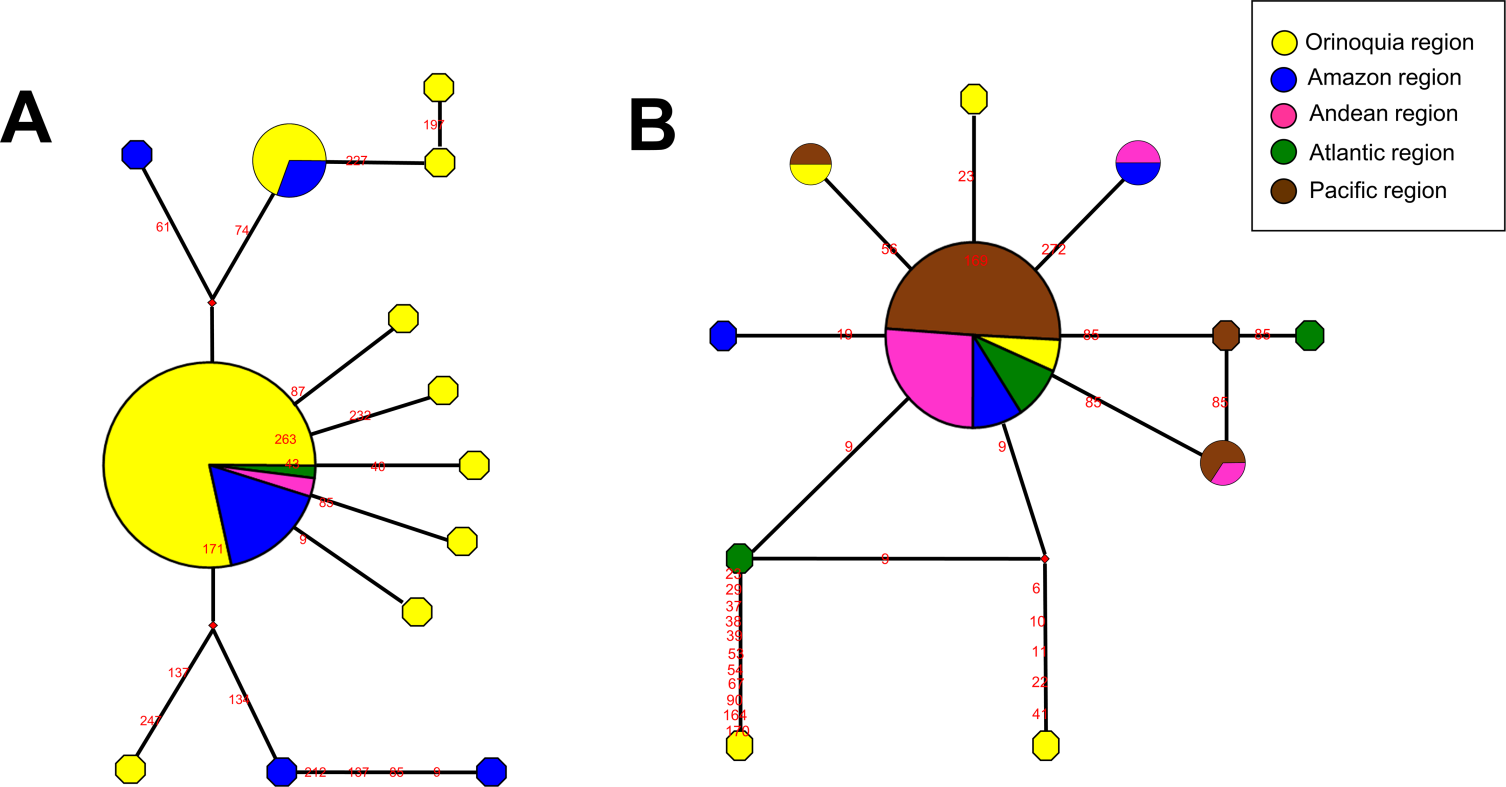
Network analysis of geographical distribution of *Leishmania* species. Alleles of the HSP70 gene were retrieved to construct the networks shown for each species as follows, the numbers on the lines specify the positions across the alignment where a nucleotide change occurred (**A**) *L*. *braziliensis* and (**B**) *L*. *panamensis*.

The haplotype network showed the high intra-specific genetic variability of *L*. *braziliensis* and *L*. *panamensis*. For *L*. *braziliensis* 13 types of sequence were identified and for *L*. *panamensis* 11, distributed in the different geographical areas analyzed. This analysis was performed only with these two species, because they were the most frequent in our study. For *L*. *braziliensis*, two of the haplotypes found were identified in more than one individual and in more than one geographical area. The most dense of these haplotypes, was distributed mainly in the Orinoquia region, followed by the Amazon region and to a lesser extent in the Andean and Atlantic regions; the second most prevalent haplotype was distributed mainly in the Orinoquia and Amazon regions. The remaining haplotypes were considered independent and distributed mainly in the Amazon and Orinoquia regions. Interestingly, we observed that the alleles of the Amazon region were shared with the alleles of the Andean region **(Fig 4A)**. Regarding *L*. *panamensis*, we found four haplotypes distributed in more than one geographical region, where the most abundant was distributed in all geographic regions analyzed in the study (Pacific, Andean, Orinoquia, Amazon and Atlantic regions). The second haplotype was distributed in the Pacific and Andean regions, and the third and fourth haplotypes distributed equally in the Andean and Amazon regions and Orinoquia and Pacific, respectively. The remaining haplotypes were found in a single individual and in a particular geographical area **(Fig 4B)**.

## Discussion

At present, it is well known that the main factor influencing the occurrence of outbreaks of Leishmaniasis, and other vector-borne diseases in the army population is the high number of personnel entering endemic areas with high circulation of the vector insect, which coincide with operational areas, due to Armed conflict in the country and the fight against drug dealing and the illegal minery. Between 2005 and 2009, approximately 45,000 cases of Leishmaniasis were reported, [4] and although the numbers have declined considerably during the years 2011 to 2017 (17.796 cases of CL and 254 cases of MCL) due to the recent Peace deal with the rebels of the FARC, the data remain alarming.[18] Previous studies have determined that the main clinical manifestation presented in the Colombian army population is CL, with ulcerative lesions considered the main forms of presentation. This work was carried out for several purposes: one of which was to expand the information currently available on the species of *Leishmania* that are circulating in areas where there is a greater military deployment and for the first time to determine the geographical distribution of these species in our country. The clinical-epidemiological analyzes carried out in the present study confirm that 97% of cases were associated with CL, and additionally, despite the use of personal protection elements to avoid insect bites (repellents and uniforms impregnated with Permethrin), the army continues to present a high infection rate; About 90% of the patients included in the present study (positive for leishmaniasis) reported using one or more of these elements. Although the 31% of patients positive for leishmaniasis mentioned having presented the disease previously, the data obtained did not allow us to determine if this was associated with resistence to treatment, reactivation or reinfection.

Although, the data reported so far in the army population correlate with the most common clinical form of the disease (CL), the information associated with the species involved is still scarce. To date, there have been several studies in which mitochondrial (Cytb) and nuclear (HSP-70) gene sequencing have been used to identify species of the genus *Leishmania*. [14, 19–22] In our case, the direct sequencing of these molecular markers was successful and sufficiently sensitive for the typing of *Leishmania* species from clinical samples. We observed that HSP70 gene was more sensitive than CytB in the detection of parasite DNA from clinical samples, due to differences in copy number and because this marker has major power resolution (64 SNPs in total) than Cytb. However, around 19% of samples positive by microscopy could not be identified. These samples were PCR negative by kDNA. This was unexpected and might be explained due to the presence of potential inhibitors of the samples or unlikely manipulation of the sample. Also, a possible mistake during the DNA extraction that did not allow the molecular detection of *Leishmania*. Lastly, it is important to highlight the limitation of the imprint that could explain the lack of congruence between microscopy and PCR results. Unfortunately, we did not have additional sample to repeat the process and rule out these assumptions. The 81% of the samples were identified showing five species of *Leishmania*, which are circulating in the areas where Colombian army personnel are deployed. *L*. *braziliensis* was the most frequently occurring species (61.1%), confirming the reported by Perez-Franco et al. 2016, where 95.4% of the 45 samples of patients with CL, belonging to the Colombian National Army were associated with *L*. *braziliensis*. [13] When analyzing a larger population group, we were able to identify other species of *Leishmania* such as *L*. *panamensis*, *L*. *guyanensis*, *L*. *mexicana* and *L lainsoni* **(Table 1)**. The latter species recently identified in the departments of Putumayo and Antioquia in Colombia.[14] In addition, the analysis of the HSP70 gene allowed the identification of different genotypes within *L*. *braziliensis* and *L*. *panamensis* **(Fig 1)** in accordance with the reported by Van der Auwera et al., 2013 and 2015 suggesting the existance of intraspecific genetic variability using this locus. [22, 23]

When comparing these results with those reported in the urban population, we observed that the proportion and species identified in rural areas are the same as those that are circulating in urban areas. [14, 24] In general, we confirm that *L*. *braziliensis* and *L*. *panamensis* are not only the species most frequently associated with CL in Colombia, as described by several authors,[14, 24, 25] but they are also the main species that are circulating more frequently in all our Colombian territory. Additionally, when we performed the analysis of species at the municipal level, we could observe that there are municipalities (mainly from the department of Meta), in which more than one species of *Leishmania* is circulating at the same time **(Fig 3B)**. Our findings have important implications, since the existence of two or more species circulating in the same geographical area increases the risk of reinfections, creates inconvenients at the moment of *Leishmania* species identification associated with the disease, in the selection of antileishmanial therapy (due to the influence of the species on the clinical outcome after treatment). [26–29] and the possibility of increasing the risk of resistance to anti-leishmanial therapy.

On the other hand, all studies conducted so far in the army population, describe the distribution of species at the geographical level but none analyzes the genetic diversity. [8, 13] So far, only one study using samples from urban population is reported.[14] Our study is the first to describe, in samples from the rural area the distribution and genetic diversity of *Leishmania* species. Herein and by sequencing the two previously described markers, we identified that *L*. *mexicana* and *L*. *guyanensis* are the most diverse species (Hd = 0.66, S = 3 and Hd = 1, S = 7, respectively) **(Table 3)**. However, we believe that these findings should be confirmed with a higher number of positive samples for these species, which was out of reach, due to the low frequency in our population. In the cases of *L*. *braziliensis* and *L*. *panamensis*, we observed that these species are equally diverse (Hd = 0.33 and 0.25 respectively for the HSP-70 gene) and (Hd = 0.962 and 0.964 respectively, for the Cytb gene) **(Table 3)**, which was confirmed by the haplotype network, in which 11 to 13 different sequence types were observed for each species **(Fig 4A and 4B)**. One limitation of these assumptions is that we only used two genetic markers to unravel the intra-species genetic diversity. However, we did not have access to the *Leishmania* isolate of the patient. Therefore, the only option was to use two sensitive markers to pull out potential and informative SNP´s for these calculations. For further studies, it is mandatory to conduct Multilocus Sequence Analyses or Whole Genome Sequencing to obtain a suitable picture of intra-species variability.

Although, our analyzes determined that *L*. *braziliensis* is the most frequently encountered species, most of the haplotypes were limited to two geographic areas in particular (Orinoquia region and Amazon region), contrary to *L*. *panamensis*, where was observed a lower frequency but a broader geographical distribution **(Fig 4A and 4B)**. These results vary regarding to what was observed until 2005 in urban areas, where *L*. *panamensis* was the species with the highest frequency of occurrence [14, 24] and *L*. *braziliensis* the species with broadest distribution.[14] Our results also allowed to determine not only the high intra-specific genetic variability of *L*. *braziliensis* and *L*. *panamensis* in the Colombian army population but also to observe how these genotypes/haplotypes are being distributed at the geographic level. One of the main reasons for the geographic shift in species and genotypes of *Leishmania* in most of the Colombian territory is due to the armed conflict in our country, which has caused an increase in the phenomena of displacement of the population towards endemic areas as well as movement of armed groups to and from these areas which has changed the epidemiology of the disease and consequently the distribution of the species. One of the species in which this phenomenon has been clearly observed is *L*. *guyanensis*, which was a prevalent species on the shores of the Orinoco and Amazon rivers[30] and in recent years has been detected in two different habitats; Both in the Andean region (Department of Tolima) [14] as in the Caribbean region (Department of Sucre) of the country.[24]

In conclusion, the results obtained from this study allowed to determine that currently five species of *Leishmania* are circulating in the areas where the Colombian army personnel are deployed, of which *L*. *braziliensis* is the species with the highest frequency of occurrence. We also determined the wide geographical distribution of these species in the national territory and identified that in some departments there is not only a high prevalence of cases but also more than one species of *Leishmania* is circulating at the same time. Likewise, this study allowed to identify the high intra-specific genetic variability of *L*. *braziliensis* and *L*. *panamensis* and how these genotypes are distributed at the geographic level.

## Supporting information

S1 Fig Distribution of *Leishmania* species at the municipal level. The diagram shows the number and percentage of cases in which each species of *Leishmania* was identified in the different municipalities.
(TIF)
