# Supplementary figures and images for: Spatial distribution, Leishmania species and clinical traits of Cutaneous Leishmaniasis cases in the Colombian army
Source: PLoS Negl Trop Dis. 2017 Aug 29;11(8):e0005876. doi: 10.1371/journal.pntd.0005876 (PMC5593196; doi:10.1371/journal.pntd.0005876)

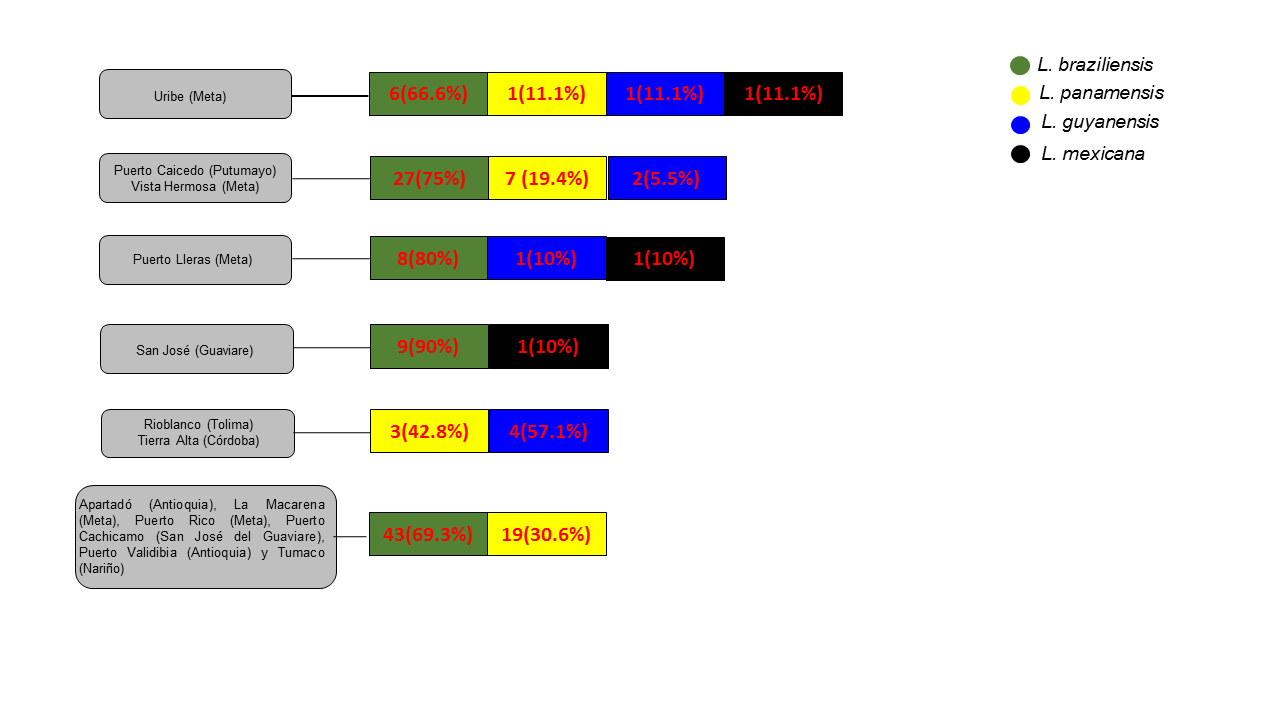

Supplement: S1 Fig — The diagram shows the number and percentage of cases in which each species of Leishmania was identified in the different municipalities. (TIF) [file pntd.0005876.s001.TIF]
